# Maintenance of *MYC* expression promotes de novo resistance to BET bromodomain inhibition in castration-resistant prostate cancer

**DOI:** 10.1038/s41598-019-40518-5

**Published:** 2019-03-07

**Authors:** Daniel J. Coleman, Lina Gao, Jacob Schwartzman, James E. Korkola, David Sampson, Daniel S. Derrick, Joshua Urrutia, Ariel Balter, Julja Burchard, Carly J. King, Kami E. Chiotti, Laura M. Heiser, Joshi J. Alumkal

**Affiliations:** 1 Knight Cancer Institute, Oregon Health & Science University, Portland, OR 97201 USA; 2 Department of Biomedical Engineering, Oregon Health & Science University, Portland, OR 97201 USA; 3 Computational Biology Program, Oregon Health & Science University, Portland, OR 97201 USA; 4 Department of Molecular and Medical Genetics, Oregon Health & Science University, Portland, OR 97201 USA

## Abstract

The BET bromodomain protein BRD4 is a chromatin reader that regulates transcription, including in cancer. In prostate cancer, specifically, the anti-tumor activity of BET bromodomain inhibition has been principally linked to suppression of androgen receptor (AR) function. *MYC* is a well-described BRD4 target gene in multiple cancer types, and prior work demonstrates that *MYC* plays an important role in promoting prostate cancer cell survival. Importantly, several BET bromodomain clinical trials are ongoing, including in prostate cancer. However, there is limited information about pharmacodynamic markers of response or mediators of de novo resistance. Using a panel of prostate cancer cell lines, we demonstrated that *MYC* suppression—rather than AR suppression—is a key determinant of BET bromodomain inhibitor sensitivity. Importantly, we determined that BRD4 was dispensable for *MYC* expression in the most resistant cell lines and that *MYC* RNAi + BET bromodomain inhibition led to additive anti-tumor activity in the most resistant cell lines. Our findings demonstrate that *MYC* suppression is an important pharmacodynamic marker of BET bromodomain inhibitor response and suggest that targeting *MYC* may be a promising therapeutic strategy to overcome de novo BET bromodomain inhibitor resistance in prostate cancer.

## Introduction

Although treatment options for patients with castration-resistant prostate cancer (CRPC) are expanding, over 29,000 American men are still predicted to die from prostate cancer in 2018^1^. This demonstrates the urgent need to develop more effective therapies to treat this disease. We and others determined that inhibition of BET bromodomain proteins represents a promising strategy to treat CRPC^2–4^. BET bromodomain proteins are chromatin readers that recognize and bind to acetylated lysines on histone tails, such as lysine 27 on Histone H3 (H3K27Ac)^5^. BET bromodomain proteins then recruit other transcriptional machinery that promote expression of important genes in cancer^6^. BET bromodomain inhibition blocks proliferation and promotes differentiation^7^, which contributes to cancer cell death. However, key markers of response are unknown, and very little is known about mechanisms of de novo resistance to BET bromodomain inhibition in CRPC.

MYC is a key transcription factor regulated by multiple cellular pathways and transcriptional regulators, including BET bromodomain proteins^5,8^. Importantly, re-activation of *MYC* mRNA expression has been implicated as an acquired resistance mechanism to BET bromodomain inhibition in several cancer types, demonstrating MYC’s importance^9–12^. Herein, using a panel of CRPC cell lines, we demonstrate that suppression of *MYC* expression by BET bromodomain inhibition strongly correlates with sensitivity to the BET bromodomain inhibitor JQ1. Further, we demonstrate that co-targeting *MYC* together with BET bromodomain inhibition in cells in which JQ1 fails to suppress *MYC* expression leads to additive anti-tumor activity. Thus, our results demonstrate the utility of measuring *MYC* expression as a pharmacodynamic marker of BET bromodomain inhibitor response and demonstrate the need to develop strategies to co-target MYC along with BET bromodomain inhibition in resistant cancer cells.

## Results

### *MYC* maintenance contributes to de novo BET bromodomain inhibitor resistance

**Figure 1:**
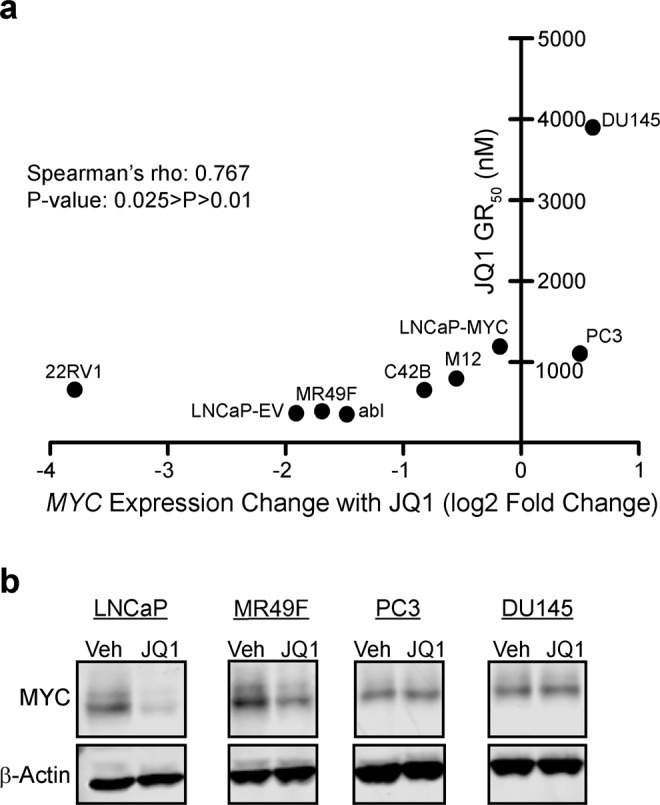
Suppression of *MYC* expression correlates with JQ1 sensitivity. (**a**) The indicated cell lines were treated with dose escalation of JQ1 or DMSO vehicle in triplicate. Plotted GR_50_ values (y-axis) are the mean of at least two independent experiments, except for M12 (see Supplementary Table S1). RNA-seq was performed on the same cell lines (one replicate per condition) treated with either DMSO or 500 nM JQ1. The log2-transformed normalized expression of *MYC* for each cell line is indicated on the x-axis. A Spearman’s Rank-Order Correlation was performed on the two datasets. (rho = 0.767, P-value is in range of 0.01–0.025 as determined by Spearman’s Rho table). (**b**) Western blot of MYC protein expression in multiple CRPC cell lines.

We first treated a panel of nine CRPC cell lines with dose-escalation of the BET bromodomain inhibitor JQ1 for 72 hours in order to determine the drug’s GR_50_ value (dose corresponding to 50% growth rate inhibition) in each cell line (Supplementary Table S1). This panel included both AR-dependent and AR-independent CRPC cell lines as well as AR-dependent LNCaP cells engineered to stably overexpress an ectopic *MYC* cDNA not under the control of BET bromodomain proteins (LNCaP-MYC). AR-independent PC3 and DU145 cells, and LNCaP-MYC cells were the least sensitive to JQ1 (Fig. 1a, y-axis). Further, LNCaP-MYC cells had a GR_50_ more than 3-fold higher than LNCaP cells overexpressing an empty control vector (LNCaP-EV, Fig. 1a, y-axis). Based on these results, we hypothesized that the degree of suppression of *MYC* expression by JQ1 may influence sensitivity to JQ1. To test this question, we examined our RNA-seq data from the same cell line panel treated +/−500 nM JQ1 for 24 hours. For each cell line, we plotted *MYC* expression change with JQ1 (Fig. 1a, x-axis) vs. GR_50_ (Fig. 1a, y-axis). Importantly, *MYC* was not repressed by JQ1 in PC3 and DU145 cells, and we observed a significant correlation between *MYC* mRNA decline by JQ1 and JQ1 sensitivity (Fig. 1a). Higher baseline *MYC* mRNA expression weakly correlated with JQ1 sensitivity, but was not significant (Fig. S1). To verify lack of *MYC* suppression by JQ1 in resistant PC3 and DU145 cells as compared to sensitive LNCaP and MR49F cells, we treated cells +/−500 nM JQ1 for 24 hours and then performed western blots to assess MYC protein levels. JQ1 treatment strongly suppressed MYC protein levels in the highly sensitive LNCaP and MR49F lines. However, JQ1 failed to suppress MYC levels in PC3 and DU145 (Fig. 1b). Altogether, our data suggests that reduction of *MYC* expression is a marker of sensitivity to BET bromodomain inhibition and that persistent *MYC* expression may confer de novo resistance.

### Confirmation of the importance of AR-independent effects for the anti-tumor activity of BET bromodomain inhibition

**Figure 2:**
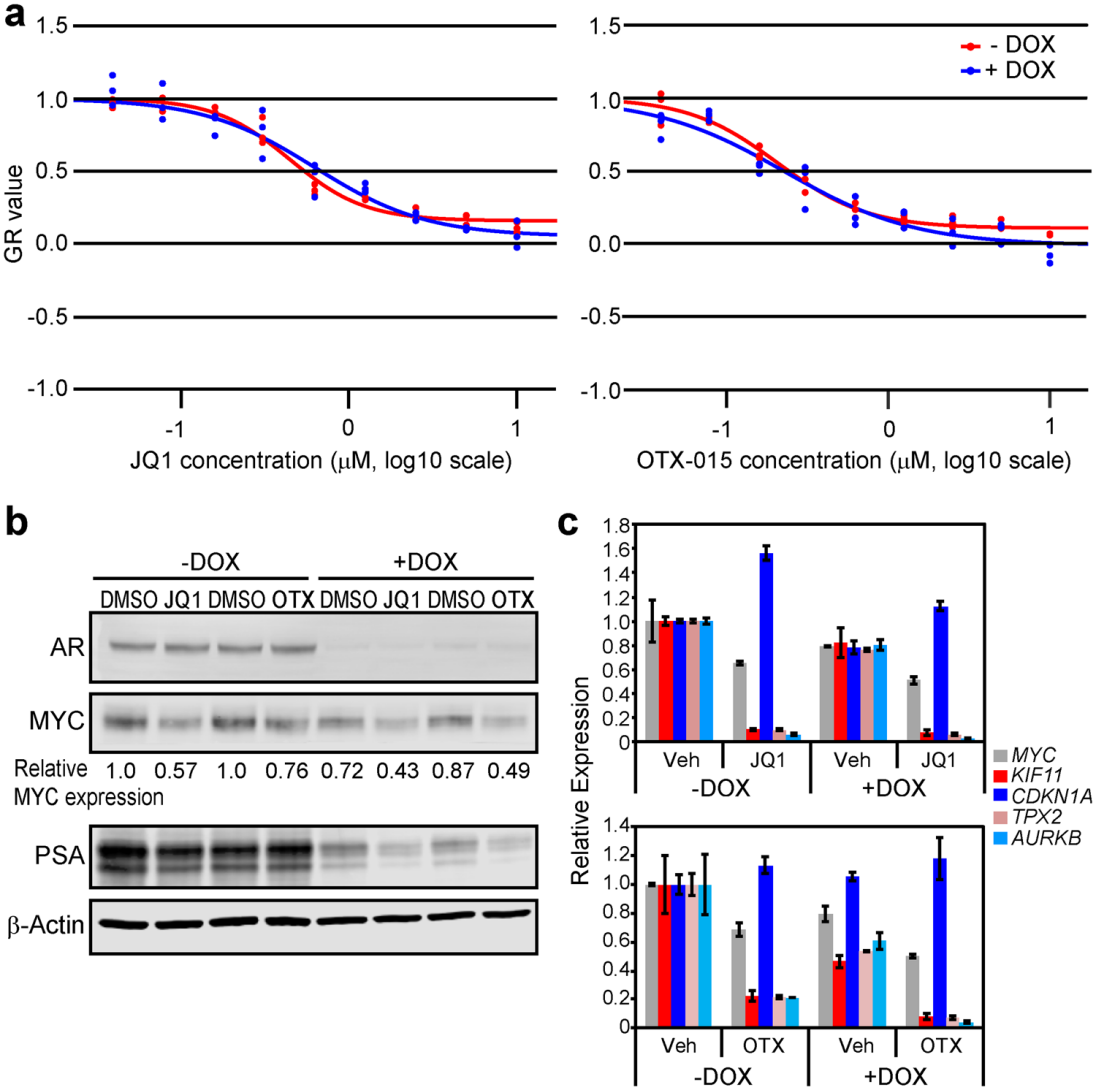
AR suppression does not abrogate the effects of BET bromodomain inhibition. (**a**) C4-2 shAR cells^13^ were plated at proper density for logarithmic growth in 96-well plates and treated the next day with dose escalation of JQ1 or OTX-015 [+/−2 µg/mL doxycycline (DOX) to knock down AR expression] in triplicate wells. Relative cell number was measured at time 0 and 72 hours later with the CellTiter-Glo 2.0 Assay Kit (Promega) in order to determine relative growth rate. Individual replicates for each dose are plotted. (**b**) Western blot of AR, MYC, and PSA protein expression in cells treated +/−DOX as in (**a**) and with 500 nM JQ1 or 230 nM OTX-015. To quantify MYC protein levels, the intensity of each band as measured by ImageJ was normalized to its corresponding β-Actin control. Relative MYC expression is represented as fold change vs. control (−DOX, DMSO). (**c**) RT-qPCR of *MYC* mRNA and MYC target gene expression in samples corresponding to (**b**). Data is mean of 2 biological replicates. Error bars represent +/−SEM.

Prior work, including our own, suggested that suppression of AR function is a key mediator of BET bromodomain inhibitor response in CRPC^3,4^. The cells most resistant to JQ1 in our panel—PC3 and DU145—are AR-independent CRPC cell lines, but our results also suggested that a lack of suppression of *MYC* expression by JQ1 in these cell lines may be more important. Therefore, to determine the importance of suppression of AR function on BET bromodomain inhibitor response, we used a CRPC cell line (C4-2 cells) engineered to express a doxycycline-inducible *AR* shRNA^13^ (C4-2 shAR). We treated the cells +/−doxycycline and subjected them to a dose range of JQ1 or OTX-015, a BET bromodomain inhibitor that has been used clinically (Fig. 2a). We confirmed that doxycycline induced AR knockdown in these cells and blocked AR function as determined by a reduction in PSA (Fig. 2b). By measuring relative growth rate of the cells after 72 h of treatment, we determined that knockdown of AR did not change the sensitivity of the cells to either BET bromodomain inhibitor (Fig. 2a).

We previously demonstrated that AR can regulate MYC expression and function in several CRPC cell lines^2^. In this current study, using the C4-2 shAR model, we found that BET bromodomain inhibition had a greater effect on reducing MYC protein levels compared to AR knockdown (Fig. 2b). Moreover, BET bromodomain inhibition was equally capable of reducing MYC levels in the AR-expressing or AR-deficient cells (Fig. 2b). We then investigated the effects of BET bromodomain inhibition and/or AR knockdown on MYC function, by measuring expression of MYC target genes identified previously in CRPC^2^. AR knockdown alone only modestly reduced MYC function, while both bromodomain inhibitors strongly reduced MYC function regardless of AR expression (Fig. 2c). Altogether, our data suggest that suppression of MYC—rather than suppression of AR—may be a key factor that determines BET bromodomain inhibitor sensitivity in CRPC.

### BET bromodomain proteins are dispensable for *MYC* expression in the cell lines most resistant to BET bromodomain inhibition

**Figure 3:**
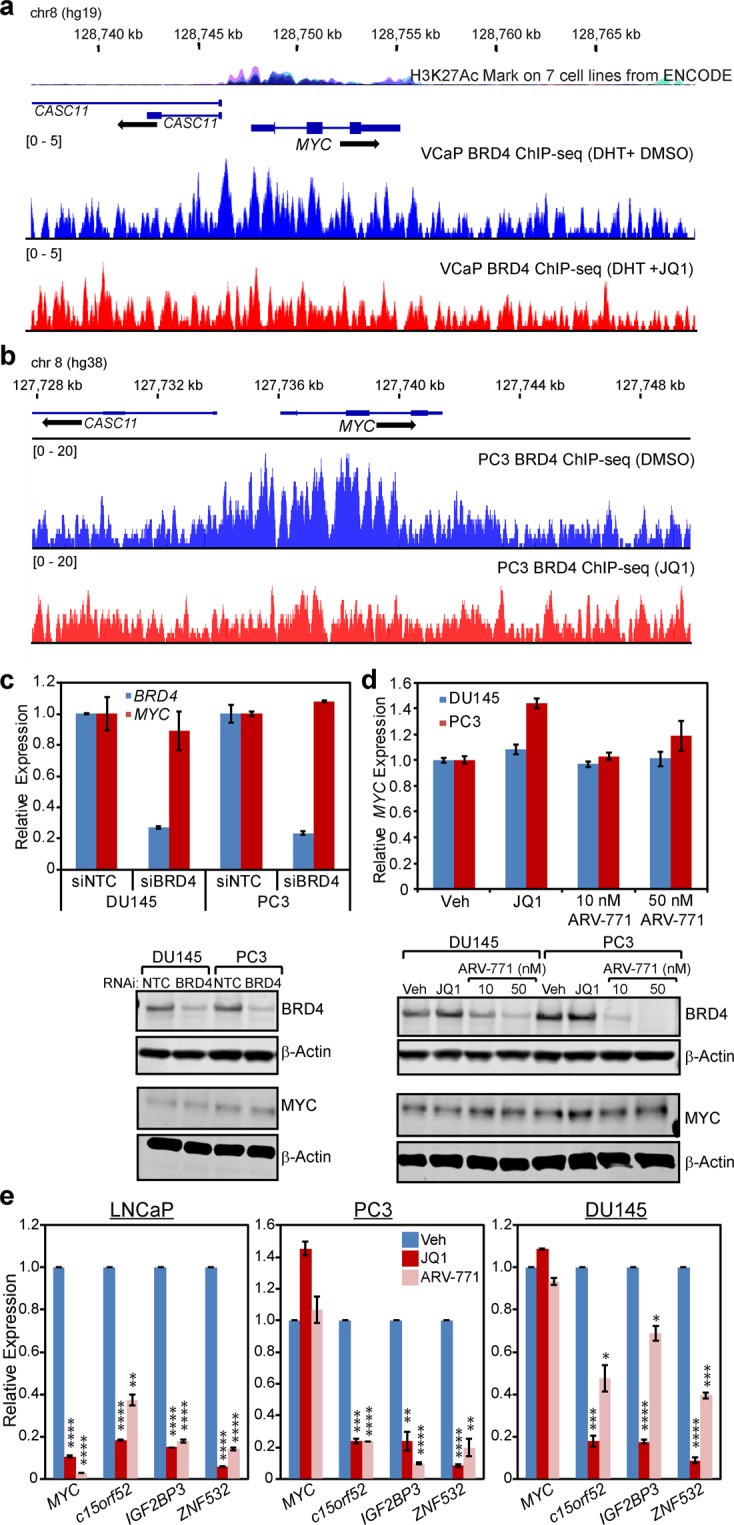
*MYC* expression is not dependent on BET bromodomain proteins in the cell lines most resistant to BET bromodomain inhibition. (**a**) ChIP-seq for BRD4 in VCaP cells treated for 12 h with dihydrotestosterone (DHT) plus either vehicle (mock) or 500 nM JQ1^4^. (**b**) ChIP-seq for BRD4 in PC3 cells treated for 12 h with either vehicle (mock) or 500 nM JQ1. BRD4 binding at the *MYC* locus is shown. Two independent ChIP-seq experiments were performed for each treatment which yielded comparable results. One representative experiment is shown. (**c**) RT-qPCR (top) and western blot (bottom) of *MYC* mRNA and MYC protein expression, respectively, in PC3 and DU145 cells transfected with either control or BRD4 siRNA. Data is mean of 2 biological replicates. Error bars represent +/−SEM. (**d**) RT-qPCR (top) and western blot (bottom) of *MYC* mRNA and MYC protein expression, respectively, in PC3 and DU145 cells treated with either JQ1 (500 nM) or the small-molecule pan-BET bromodomain protein degrader ARV-771^16^. Data is mean of 2 biological replicates. Error bars represent +/−SEM. (**e**) RT-qPCR of mRNA expression of *MYC* and several other BET bromodomain target genes in LNCaP, PC3, and DU145 cells treated with vehicle, JQ1 (500 nM) or ARV-771 (50 nM). Data is mean of 2 biological replicates. Error bars represent +/−SEM. *p ≤ 0.05, **p ≤ 0.01, ***p ≤ 0.001, ****p ≤ 0.0001 (two-tailed t-test).

Because we observed that *MYC* was not repressed by JQ1 in PC3 and DU145 cells, we next sought to confirm that BET bromodomain proteins were not important for regulating *MYC* expression in these cells. First,we examined published ChIP-seq data in the VCaP CRPC cell line^4^, in which *MYC* expression was shown to decrease with JQ1 treatment^4^. This analysis demonstrated that BRD4 is enriched at the *MYC* locus in VCaP cells and that BRD4 binding is reduced by JQ1 (Fig. 3a). The region of BRD4 enrichment is consistent with ChIP-seq data from ENCODE that shows elevated H3K27Ac in a panel of seven cell lines (Fig. 3a). Next, we performed ChIP-seq of BRD4 in PC3 cells treated +/−JQ1. Interestingly, we also observed a significant enrichment of BRD4 at the *MYC* locus in this cell line, which was also depleted by JQ1 treatment (Fig. 3b). That BRD4 depletion from this locus in PC3 did not reduce *MYC* expression suggests that other regulatory elements and factors may be more important for *MYC* expression in PC3 cells.

JQ1 interferes with binding of the bromodomain region of BET bromodomain protein family members such as BRD4 to chromatin^7^. However, BET bromodomain proteins also have bromodomain domain-independent functions that contribute to gene expression^14,15^. Therefore, to determine whether BET bromodomain proteins regulate *MYC* independently of their bromodomain domain-dependent function, we used RNAi to deplete BRD4 (Fig. 3c) or treated cells with the pan-BET bromodomain protein degrader ARV-771^16^ (Fig. 3d). Importantly, neither method of BRD4 protein depletion reduced levels of *MYC* mRNA or MYC protein in either cell line. We verified that ARV-771 was effective in reducing MYC protein levels in LNCaP cells at the same doses (Fig. S2). To confirm on-target effects of JQ1 and ARV-771 in PC3 and DU145 cells versus more sensitive cells (LNCaP), we measured expression of genes from our RNA-seq data that were similarly downregulated by JQ1 in all three cell lines. These genes (*c15orf52, IGF2BP3*, and *ZNF532*) were chosen because BRD4 was bound to their promoter regions in our PC3 ChIP-seq, and this binding was depleted by JQ1 (Fig. S3). Expression of *c15orf52*, *IGF2BP3*, and *ZNF532* were significantly reduced by JQ1 and ARV-771 in LNCaP, PC3, and DU145. In contrast, *MYC* was only reduced in LNCaP (Fig. 3e). Altogether our data demonstrate that BET bromodomain proteins are not required for transcription of *MYC* in JQ1-resistant PC3 and DU145 cell lines.

### Combination of *MYC* suppression and JQ1 treatment results in additive anti-tumor activity in the cell lines most resistant to BET bromodomain inhibition

**Figure 4:**
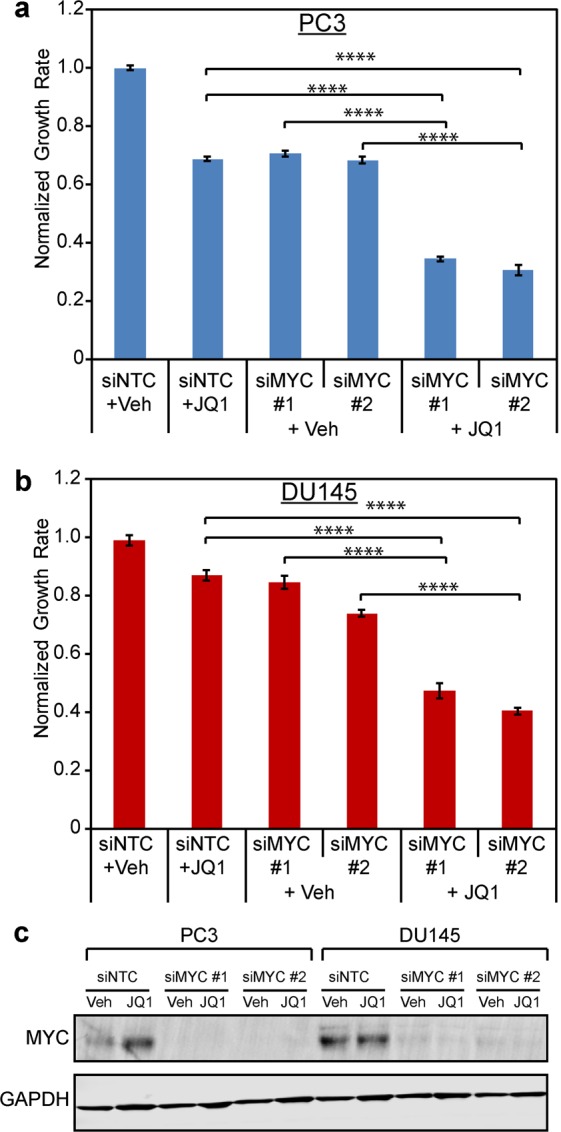
Co-suppression of MYC with BET bromodomain inhibition has additive anti-tumor activity in the cell lines most resistant to BET bromodomain inhibition. (**a**) PC3 and (**b**) DU145 cells were transfected with control (siNTC) or *MYC* siRNAs (siMYC) and treated with 500 nM JQ1 or vehicle. Relative cell number was measured at time 0 and 96 hours later with the CellTiter-Glo 2.0 Assay Kit (Promega). These assays were performed in 10 biological replicates per condition. ****p ≤ 0.0001 (one-way ANOVA with Bonferroni correction). (**c**) Western blot analysis confirming MYC protein suppression in panels a and b.

Our data demonstrate that persistent *MYC* expression confers de novo resistance to BET bromodomain inhibition. Therefore, we next sought to determine if combining *MYC* suppression with JQ1 treatment would lead to additive anti-tumor activity in cells in which JQ1 failed to suppress *MYC*. To determine that, we used RNAi to deplete *MYC* in PC3 and DU145 cells and then treated the cells +/−500 nM JQ1 for 96 hours (Fig. 4). JQ1 treatment or *MYC* knockdown alone had modest effects on growth rate compared to cells transfected with non-targeted control siRNA and vehicle (Fig. 4). However, combined *MYC* RNAi + JQ1 treatment led to a significant additive effect on suppressing growth of both cell lines. Importantly, this effect was recapitulated with multiple *MYC*-targeting siRNAs (Fig. 4). These data demonstrate that maintenance of *MYC* is not only a marker of resistance to BET bromodomain inhibition but also that co-targeting MYC together with BET bromodomain inhibition may be a useful strategy to overcome de novo resistance.

## Discussion

BET bromodomain inhibition is a promising therapeutic strategy for cancer, and there are several recently completed or ongoing BET bromodomain inhibitor clinical trials, including in prostate cancer (NCT02259114, NCT02711956, NCT02705469, NCT02391480). However, there is a dearth of information on biomarkers of response. Numerous pre-clinical studies in a wide array of cancer types have identified *MYC* as a key target of BET bromodomain inhibition^5,8,17,18^, and our results corroborate those findings. Importantly, while JQ1 treatment suppressed *MYC* mRNA in most CRPC cell lines, JQ1 failed to suppress *MYC* in the most resistant cell lines. These data suggest that serial measurements of *MYC* expression in tumor cells may serve as a useful biomarker of response to BET bromodomain inhibition in the clinic.

Suppression of AR function has been previously described as a key mechanism contributing to the anti-tumor activity of BET bromodomain inhibitors in CRPC^3,4^. Importantly, the two cell lines in our panel that were least sensitive to JQ1 were AR-independent CRPC cell lines (PC3 and DU145). However, it is noteworthy that AR-independent M12 cells had a GR_50_ similar to the more sensitive AR-expressing lines. Additionally, knockdown of AR in C4-2 shAR cells did not alter their sensitivity to BET bromodomain inhibition. We previously demonstrated that AR has a role in regulating MYC expression^2^. However, AR knockdown in C4-2 cells did not impact MYC levels as strongly as BET bromodomain inhibition, and BET bromodomain inhibition reduced MYC expression even when AR was absent from these cells. Thus, it is clear that BET bromodomain proteins regulate MYC via AR-independent mechanisms in these CRPC cells. Altogether, our data suggests that targets other than the AR—such as MYC—may be important determinants of response to BET bromodomain inhibition in CRPC. This is especially important since AR-independent forms of prostate cancer may be increasing in frequency^19^, and a recent report demonstrated that MYC was an essential gene for driving progression of normal basal prostatic epithelial cells to AR-independent, neuroendocrine tumors that have features similar to clinical neuroendocrine prostate cancer^20^.

We determined that treatment of PC3 cells with JQ1 does displace BRD4 from the chromatin and suppresses expression of several BRD4 target genes, demonstrating target engagement in these cell lines. However, BRD4 eviction was not sufficient to reduce *MYC* expression. Furthermore, we confirmed that BET bromodomain protein depletion using RNAi or with the pan-BET bromodomain protein degrader ARV-771 was insufficient to reduce expression of *MYC* in PC3 and DU145 cells. Interestingly, JQ1 treatment increased MYC levels in PC3 cells, which has been observed in this cell line previously^4^. MYC upregulation as a result of JQ1 treatment has also been observed in cell line models of other cancer types, including lung cancer^21^. It is possible that BET bromodomain inhibition activates pathways that promote *MYC* expression or stability in these cell lines. Altogether, our data strongly suggest that BET bromodomain proteins are not required for activation of *MYC* mRNA expression in BET bromodomain inhibitor resistant CRPC cells such as PC3 and DU145. However, that RNAi-mediated knockdown of *MYC* combined with JQ1 led to additive anti-tumor activity in PC3 and DU145 cells, demonstrates MYC’s importance to growth of these JQ1-resistant cells.

Altogether, our findings support the utility of *MYC* as an important pharmacodynamic marker of BET bromodomain inhibitor treatment response in CRPC. Further, our work demonstrates that targeting other signaling pathways that regulate *MYC*^22–24^, or targeting MYC, itself, may be a promising approach to overcome de novo BET bromodomain inhibitor resistance and to treat MYC-driven cancers. Historically, MYC has been regarded as “undruggable.” However investigation of MYC-targeting strategies remains an extremely active area of current research^25–29^. Our results demonstrate the potential application of those agents to overcome de novo resistance to BET bromodomain inhibition in CRPC.

## Methods

### Cell Culture

LNCaP (clone FGC), 22RV1, PC3, and DU145 cells were purchased from American Type Culture Collection (ATCC, Manassas, VA). C4-2B cells were purchased from ViroMed Laboratories. Abl^30^ cells were a kind gift from Dr. Zoran Culig (Department of Urology, Innsbruck Medical University). M12 cells^31^ were a kind gift from Dr. Stephen Plymate (University of Washington Medicine). MR49F^32,33^ cells were a kind gift from Dr. Martin Gleave (Vancouver Prostate Centre and Dept. of Urologic Sciences, University of British Columbia). To generate stable *MYC* overexpressing cells, we transfected LNCaP-FGC cells with pcDNA3-2XFLAG-cMYC plasmid^34^ (a kind gift from Dr. Mu-Shui Dai, OHSU Molecular & Medical Genetics) or empty pcDNA3 vector with Lipofectamine 2000 (Life Technologies) and selected with 300 µg/mL G418 (Sigma-Aldrich). C4-2 shAR cells^13^ were a kind gift from Dr. Paul Rennie (Vancouver Prostate Centre and Dept. of Urologic Sciences, University of British Columbia). LNCaP (FGC parental, EV, and MYC), 22RV1, C42B, PC3, DU145, MR49F, and C4-2 shAR cells were grown in RPMI1640 + 10% FBS (Premium Select grade, Atlanta Biologicals). Enzalutamide-resistant MR49F cells were cultivated in media containing 10 µM Enzalutamide (MedchemExpress #HY-70002). C4-2 shAR cells were supplemented with 1 µg/mL puromycin (Sigma-Aldrich #P8833) and 1.25 µg/mL blasticidin (Sigma-Aldrich #15205) to maintain integration of the stable shRNA construct. These cells were cultured without antibiotic selection for all experiments. Abl cells were grown in RPMI with 10% charcoal/dextran-treated FBS (Atlanta Biologicals). M12 cells were grown in RPMI 1640 + 5% FBS, 0.01 µM dexamethasone (MP Biomedicals #ICN19456125), 10 ng/ml EGF (Corning #CB40052), and 10 mL/L ITS (Corning #25-800-CR). LNCaP-FGC, 22RV1, C42B, PC3, and DU145 cells were authenticated by Short Tandem Repeat (STR) profiling (DDC Medical). Other cell lines were not authenticated due to a lack of published ATCC STR profile. Mycoplasma testing was regularly performed on cells using a DNA-based PCR test^35^ and were found to be negative in all cases.

### Dose-response curve experiments

Cells were plated at proper density for logarithmic growth in 96-well plates and treated the next day with serial dilutions of JQ1 (BPS Bioscience #27402) or OTX-015 (Selleck S7360), in addition to DMSO vehicle, in triplicate wells. Cell viability was measured at time 0 and 72 hours post-treatment using the CellTiter-Glo 2.0 (CTG) assay (Promega) per manufacturer’s instructions. Nonlinear regression curves were fitted and GR_50_ values were calculated using the GRMetrics package in R^36^.

### RNA-seq library preparation and data processing

All cell lines were treated with 500 nM JQ1, and cells were harvested after 24 hours. Total RNA was extracted with Trizol/CHCl_3_ (Life Technologies). Library preparation for RNA-seq was performed as previously described^3^. Libraries were sequenced on an Illumina HiSeq as single-end 50 bp reads. RNA-seq data analysis was performed using the Tuxedo Suite^37–39^. Each sample was mapped independently to the human genome build GRCh37/hg19 using TopHat v2.0.9^37,38^. Transcript assembly and quantification was done with HTSeq v0.6.1^40^ to generate a gene-level read count value.

### Drug treatments

For all *in vitro* experiments using JQ1 (excluding dose-response curves), DMSO stock of 1 mM JQ1 was diluted 1:2000 to 500 nM in cell culture media. For *in vitro* experiments using OTX-015 (excluding dose-response curves), DMSO stock of 230 µM OTX-015 was diluted 1:1000 to 230 nM in cell culture media.100 µM and 20 µM DMSO stocks of ARV-771 (Arvinas, LLC)^16^ were diluted 1:2000 in cell culture media to 50 nM and 10 nM, respectively. Vehicle-only controls were used for all experiments, and vehicle concentrations were normalized across treatments for each experiment.

### RNAi experiments

Transient knockdowns were performed using the following siRNAs: non-targeted control (custom synthesis, 5′-CGUACGCGGAAUACUUCGAdTdT-3′), BRD4 (Dharmacon L-004937-00), MYC #1 (Ambion #4392421), MYC #2 (Dharmacon #J003282-26). Cells were transfected with DharmaFECT 3 (GE Dharmacon) transfection reagent. siRNA was transfected at a concentration of 15 nM for experiments depicted in Fig. 4, and 50 nM for experiments depicted in Fig. 3c. Cells used for RNA and protein harvest were seeded/transfected in either 12 or 6-well plates. Cells used in viability assays were seeded/transfected in 96-well plates. Cell viability was measured at time 0 and endpoint using the CTG assay described above, these values were used to calculate relative growth rate as described previously^36^.

### RNA preparation and RT-qPCR

We performed RNA preparation and RT-qPCR as described previously^3^. Briefly, RNA was extracted as described above and reverse-transcribed into cDNA. Quantitative reverse transcription PCR (RT-qPCR) was performed using TaqMan probes/reagents and a QuantStudio 3 thermocycler (Thermo Fisher). TaqMan probes were used to detect human *BRD4* (Hs04188087_m1), *MYC* (Hs00153408_m1), *c15orf52* (Hs05010185_g1), *IGF2BP3* (Hs00559907_g1), *KIF11* (Hs00189698_m1), *CDKN1A* (Hs00355782_m1), *TPX2* (Hs00201616_m1), *AURKB* (Hs00177782_m1), and *ZNF532* (Hs01557225_g1). Human *UBC* TaqMan probe (Hs00824723_m1) was used as an endogenous control. Ct data was analyzed with QuantStudio Design & Analysis Software 1.3.1 and DataAssist Software v3.0 (Thermo Fisher).

### Immunoblotting

Immunoblotting experiments were performed by running protein lysates on NuPAGE Protein gels (Thermo Fisher) and transferring onto PVDF membranes. 4–12% Bis-Tris gels were used for data in Figs 1b and 2b, 3–8% Tris-Acetate gels were used for Figs 3c,d, and S2. Antibodies used were: MYC (Abcam #ab32072), β-actin (Sigma-Aldrich #A5441), AR (Millipore 06-680), PSA (Abcam #ab53774), BRD4 (Bethyl #A301-985A) and GAPDH (Santa Cruz #sc-32233). Blots were imaged using an Odyssey imaging system (LI-COR) according to the manufacturer’s instructions.

### Chromatin immunoprecipitation (ChIP)

ENCODE data for H3K27Ac depicted in Fig. 3a was obtained as a publicly-available track on the UCSC Genome Browser. BRD4 ChIP-seq data depicted in Fig. 3a is from a publically-available dataset originally described previously^4^. ChIP-seq data depicted in Figs 3b and S3 were generated by our group using the following methods: ChIP experiments were performed with formaldehyde cross-linked cells using anti-BRD4 (Bethyl #A301-985A) or normal rabbit IgG (Millipore #12-370) antibodies. Input and ChIP samples were processed using Diagenode iDeal ChIP-seq for Transcription Factors Kit. Library preparation for ChIP-seq samples (BRD4 ChIP and input, two biological replicates per treatment) was performed using the Illumina TruSeq ChIP Sample Prep Kit, using 10 ng of input material. The samples were then sequenced using an Illumina NextSeq. 500 sequencing system. Sequences were trimmed to 100 bp, and sequencing quality was confirmed using the FastQC utility^41^. FASTQ files were aligned to the full Ensembl hg38 human genome release 85^42^ using the *bwa* utility with the *bwa-mem* algorithm^43^, and subsequently converted to sorted.bam files using the *samtools view* and *samtools sort* utilities^44^. The sequencing quality was further confirmed at this point using *samtools stats*^44^. For later analysis, output.bam files were translated to sorted.bed files using *bedtools*^45^. Peaks were called using the *epic* peak caller^46^. *Epic* is a reimplementation of the SICER algorithm^47^ which was designed to capture broad peaks associated with histone marks. For peaks called in Fig. S3, the JQ1-treated BRD4 ChIP served as the negative control in the analysis. This method allowed us to perform differential analysis by determining regions in which BRD4 is enriched in the mock-treated cells and depleted by JQ1.

### Statistical Methods

P-values for experiments depicted in Figs 1a and S1 were determined using the Spearman Rank-Order Correlation method and Spearman’s Rho table. P-values depicted in Fig. 3e were determined using a 2-tailed t-test. P-values depicted in Fig. 4a,b were determined using a one-way ANOVA with Bonferroni correction. Values for n are indicated in the corresponding figure legends for these data.

### Data Availability

RNA-seq and ChIP-seq data depicted in Figs 1a and 3b and Supplementary Figs S1 and S3 were generated by our group and deposited to the Gene Expression Omnibus (GEO) with accession number GSE98069. ChIP-seq data depicted in Fig. 3a is from a publically-available dataset originally described previously^4^. BigWig (.bw) tracks were obtained via GEO, accession GSE55062.

## Supplementary information

Supplementary Material (Figures S1-S3, full blot images)

Supplementary Table S1 - dose-response curve metrics
