# Supplementary material for: Maintenance of MYC expression promotes de novo resistance to BET bromodomain inhibition in castration-resistant prostate cancer
Source: Sci Rep. 2019 Mar 7;9:3823. doi: 10.1038/s41598-019-40518-5 (PMC6405739; doi:10.1038/s41598-019-40518-5)
Supplement: Supplementary file 1 — Supplementary Material (Figures S1-S3, full blot images) [file 41598_2019_40518_MOESM1_ESM.pdf]

Manuscript: Maintenance of *MYC* expression promotes de novo resistance to BET bromodomain inhibition in castration-resistant prostate cancer

Authors: Daniel J. Coleman, Lina Gao, Jacob Schwartzman, James E. Korkola, David Sampson, Daniel S. Derrick, Joshua Urrutia, Ariel Balter, Julja Burchard, Carly J. King, Kami E. Chiotti, Laura M. Heiser, Joshi J. Alumkal

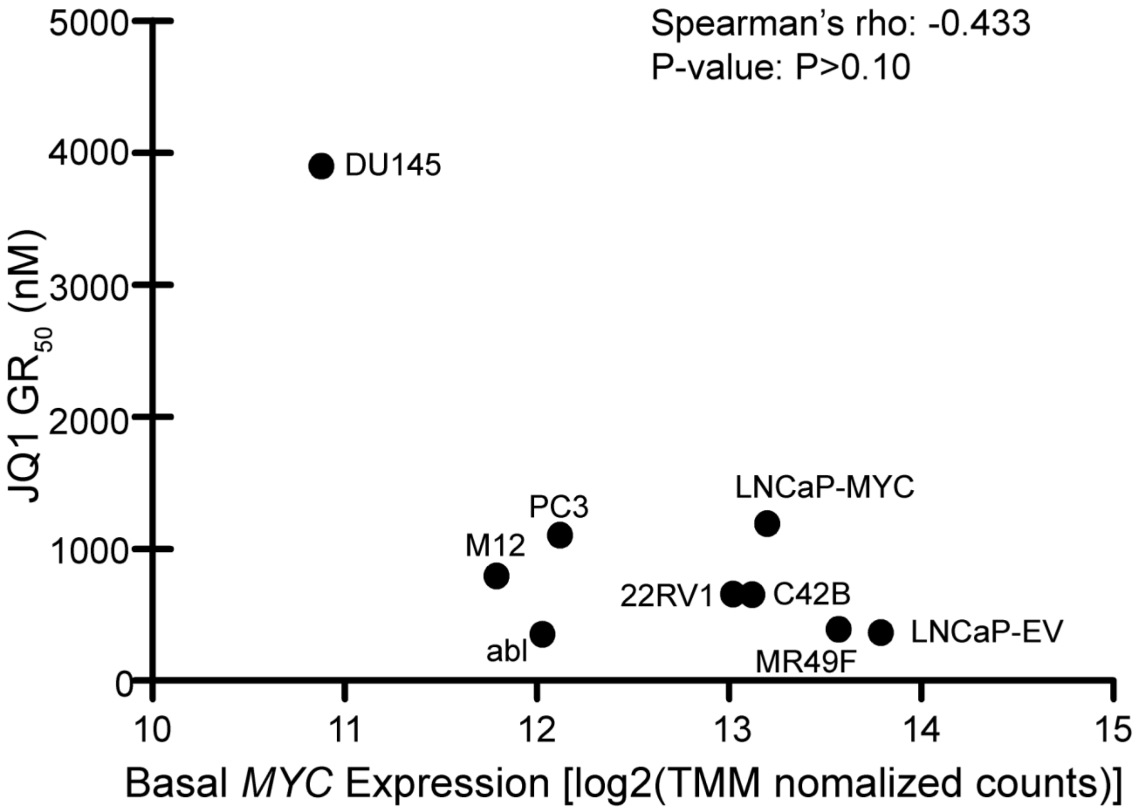

Supplementary Figure S1. Correlation of basal *MYC* expression with sensitivity to JQ1. The indicated cell lines were treated with dose escalations of JQ1, in addition to DMSO vehicle in triplicate. Plotted values are the mean of at least two independent experiments, except for M12 (see Supplementary Table S1). RNA-seq was performed on the same cell lines. The log2-TMM-normalized counts[1] for *MYC* in the vehicle treatment of each cell line is indicated on the X-axis. A Spearman's Rank-Order Correlation was performed on the two datasets. (rho= -0.433, P>0.10 (Spearman's Rho Table)).

1.) Robinson, M. D. & Oshlack, A. A scaling normalization method for differential expression analysis of RNA-seq data. Genome biology 11, R25 (2010).

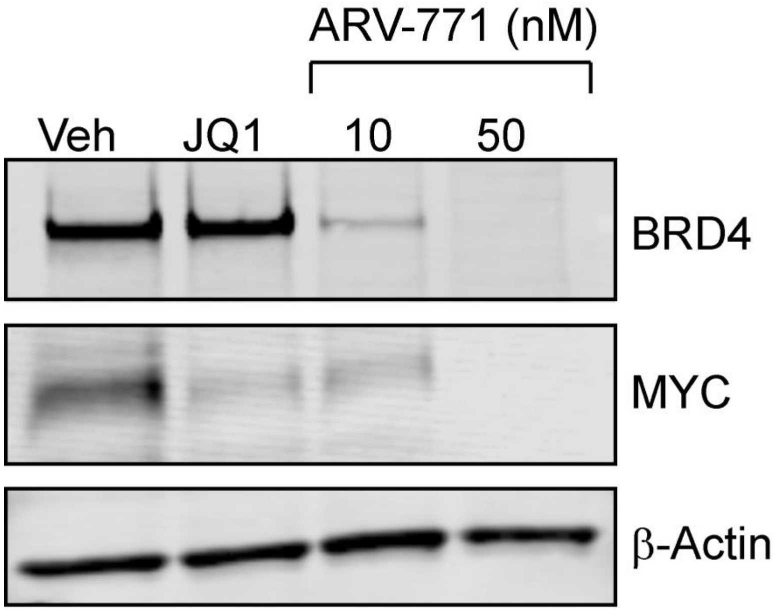

Supplementary Figure S2. MYC protein is suppressed in LNCaP cells by treatment with JQ1 or the pan-BET bromodomain protein degrader ARV-771. Western blot data from LNCaP cells treated with either JQ1 or ARV-771.

Supplementary Figure S3. Genes downregulated by BET bromodomain inhibition in LNCaP, PC3, and DU145 cells are direct BRD4 targets. ChIP-seq for BRD4 in PC3 cells treated for 12h with either vehicle (mock) or 500 nM JQ1. BRD4 binding sites at the promoter regions of *c15orf52*, *IGF2BP3*, and *ZNF532* are shown. Numbers corresponding to called peaks (horizontal black bars) represents log2 fold enrichment of BRD4 in the mock treatment vs. the JQ1 treatment at the region of the called peak. Two independent ChIP-seq experiments were performed for each treatment which yielded comparable results, one representative experiment is shown.

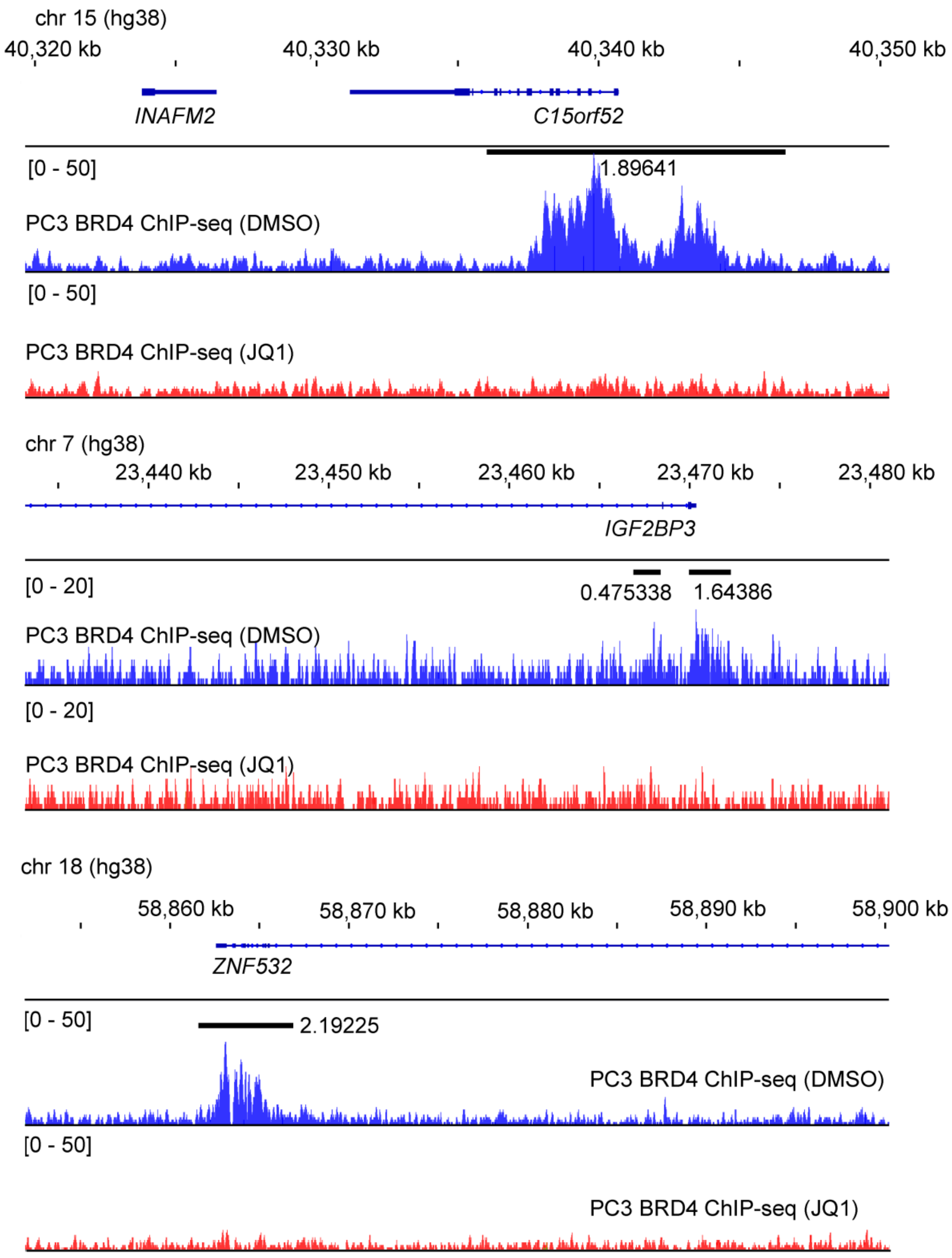

Full blot Figure 1b
Imaged with Li-Cor Odyssey CLx
Image converted to greyscale for figure

Yellow boxes indicate lanes cropped for display in figure

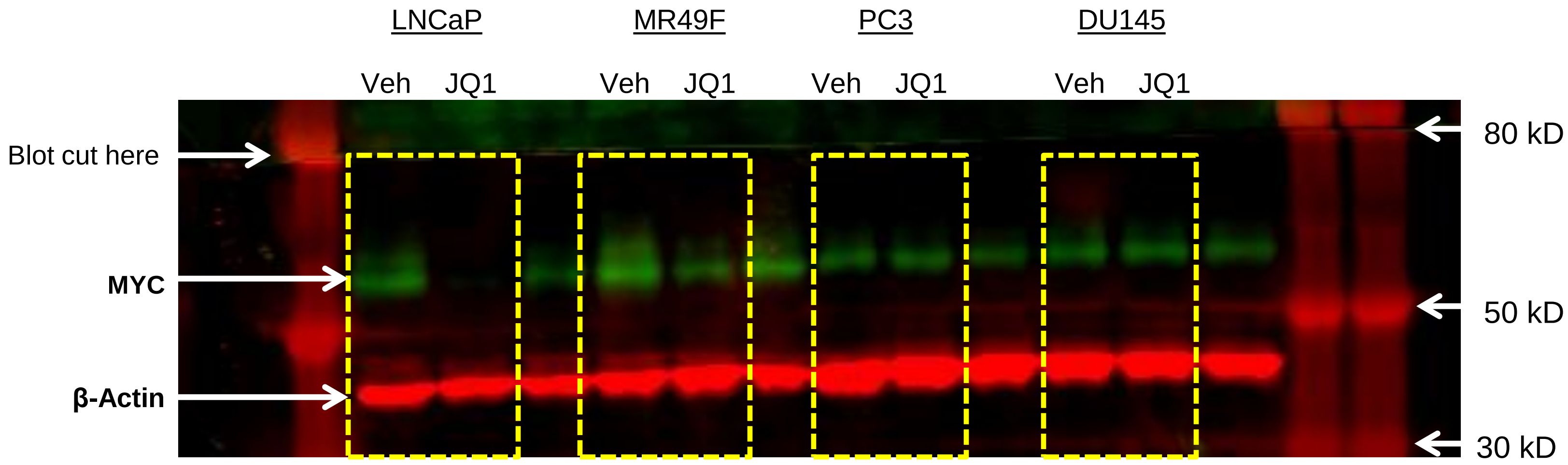

MYC imaged using Li-Cor IRDye 800CW Goat anti-Rabbit 2˚ Antibody

β-Actin imaged using Li-Cor IRDye 680RD Goat anti-Mouse 2˚ Antibody

Full blot Figure 2b
Imaged with Li-Cor Odyssey CLx
Image converted to greyscale for Figure

AR, MYC, and PSA imaged using Li-Cor IRDye 800CW Goat anti-Rabbit 2˚ Antibody

β-Actin imaged using Li-Cor IRDye 680RD Goat anti-Mouse 2˚ Antibody

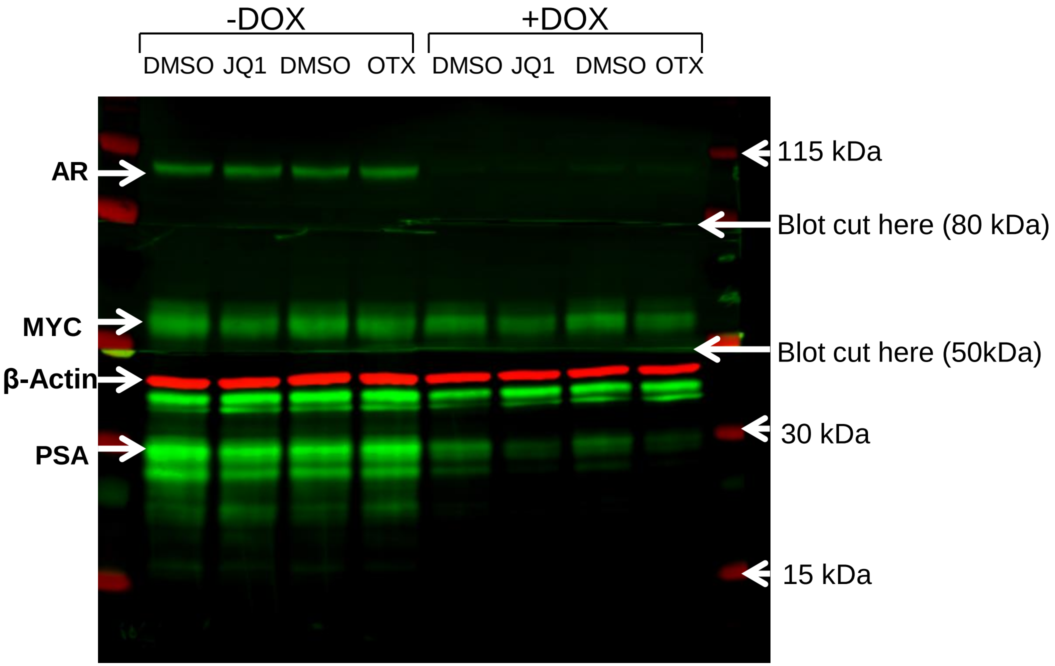

MYC re-imaged at lower exposure for Figure

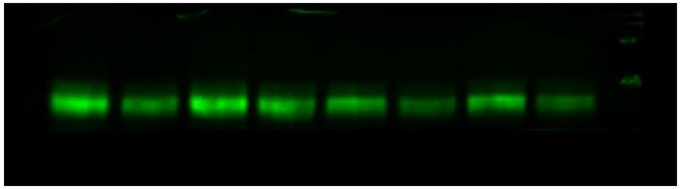

Full blots Figure 3
Imaged with Li-Cor Odyssey CLx
Images converted to greyscale for Figure
Samples were run 2x on separate gels

BRD4 and MYC imaged using Li-Cor IRDye 800CW Goat anti-Rabbit 2˚ Antibody

β-Actin imaged using Li-Cor IRDye 680RD Goat anti-Mouse 2˚ Antibody

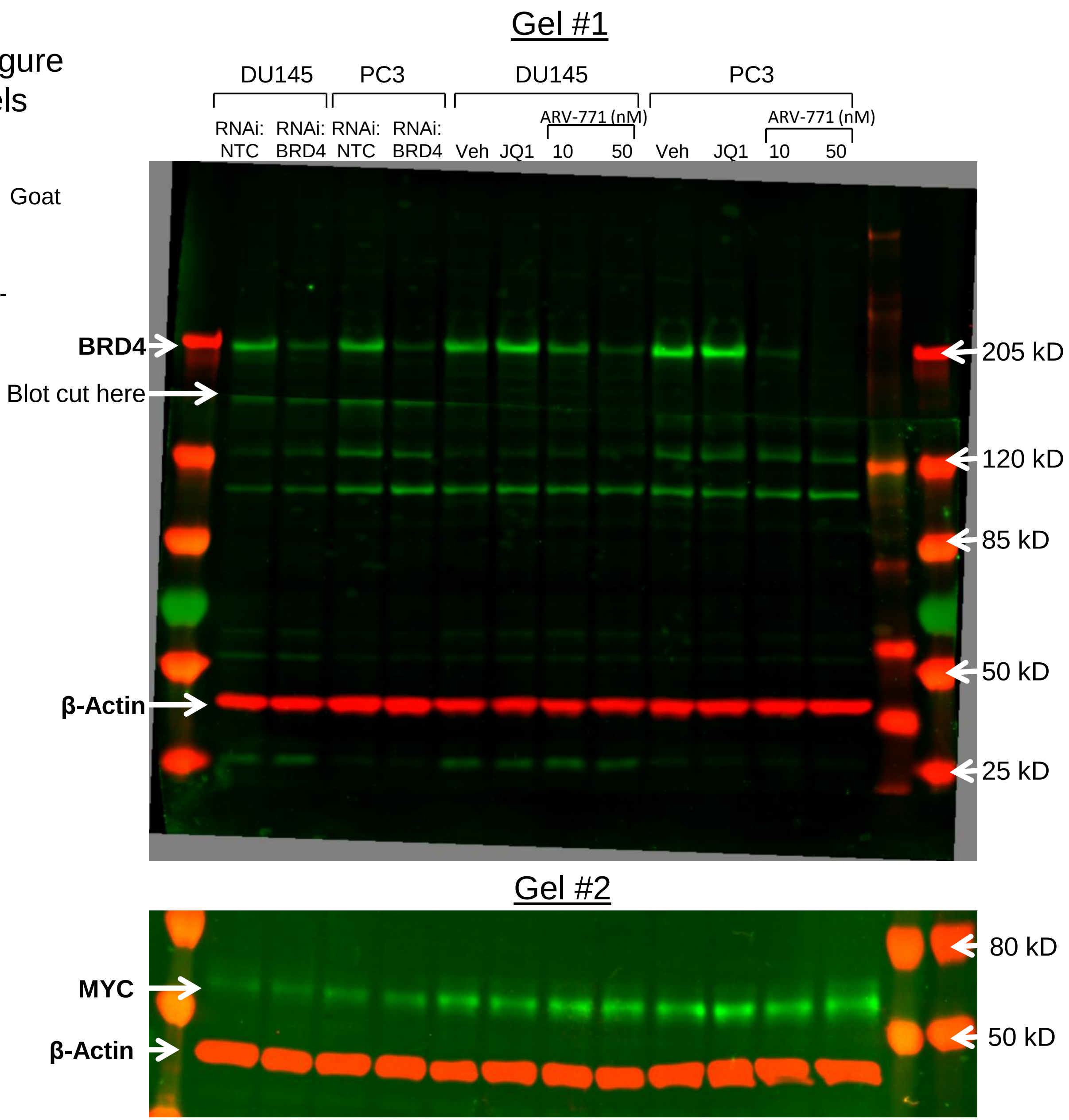

Full blot Figure S2
Imaged with Li-Cor Odyssey CLx
Images converted to greyscale for Figure

BRD4 and MYC imaged using Li-Cor IRDye 800CW Goat anti-Rabbit 2˚ Antibody

β-Actin imaged using Li-Cor IRDye 680RD Goat anti-Mouse 2˚ Antibody

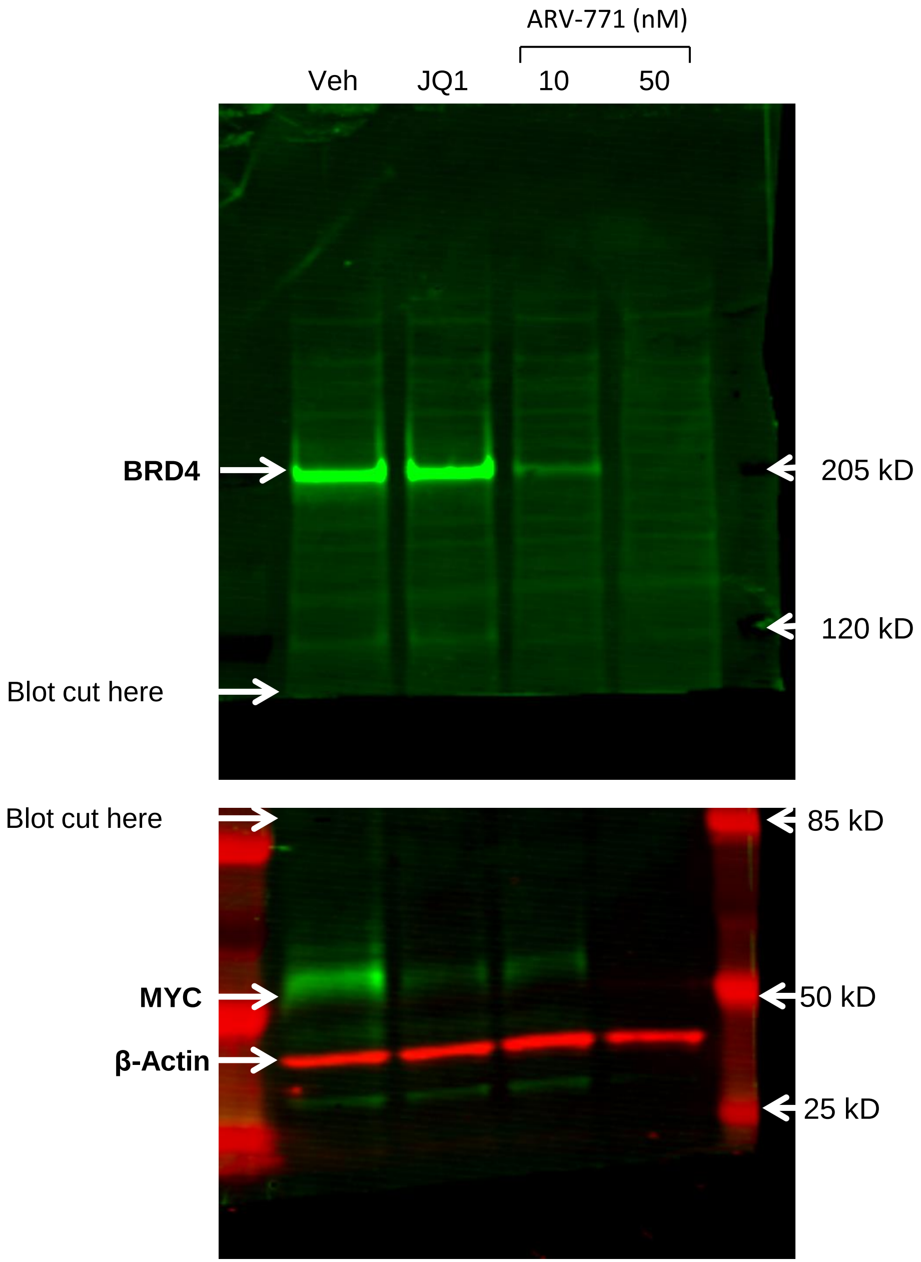

Full blot Figure 4c
Imaged with Li-Cor Odyssey CLx
Image converted to greyscale for Figure

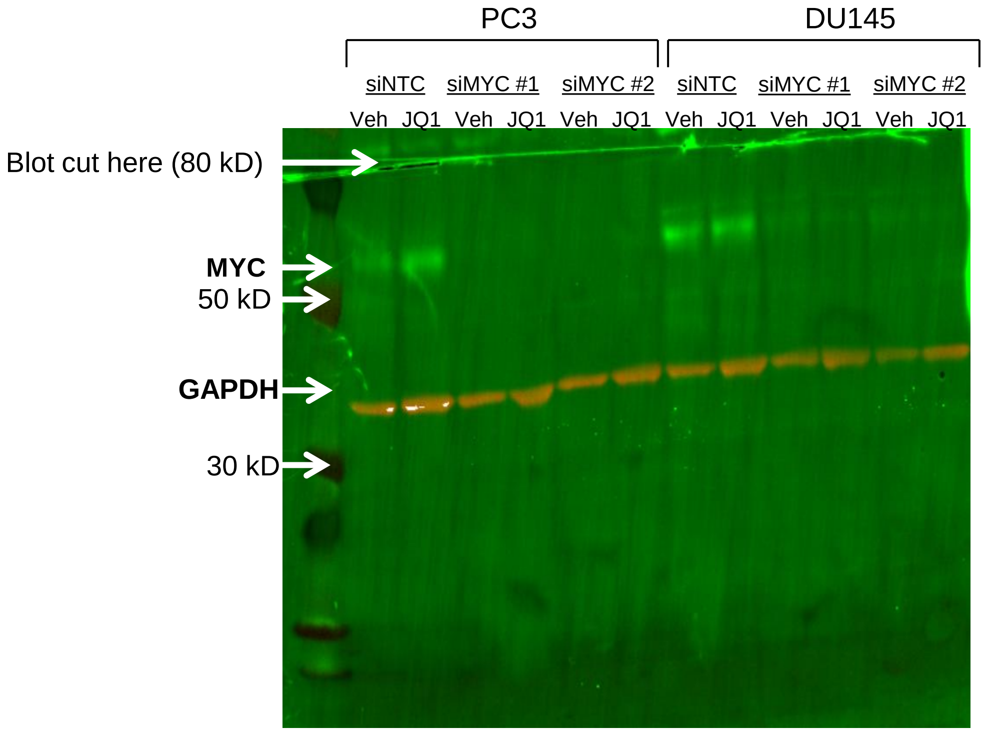

MYC imaged using Li-Cor IRDye 800CW Goat anti-Rabbit 2˚ Antibody

GAPDH imaged using Li-Cor IRDye 680RD Goat anti-Mouse 2˚ Antibody
